# High-Throughput Screening Identifies Kinase Inhibitors That Increase Dual Adeno-Associated Viral Vector Transduction *In Vitro* and in Mouse Retina

**DOI:** 10.1089/hum.2017.220

**Published:** 2018-08-01

**Authors:** Andrea Maddalena, Fabio Dell'Aquila, Pia Giovannelli, Paola Tiberi, Luca Giorgio Wanderlingh, Sandro Montefusco, Patrizia Tornabene, Carolina Iodice, Feliciano Visconte, Annamaria Carissimo, Diego Luis Medina, Gabriella Castoria, Alberto Auricchio

**Affiliations:** ^1^ Telethon Institute of Genetics and Medicine (TIGEM), Pozzuoli, Italy.; ^2^ Department of Precision Medicine, University of Campania Luigi Vanvitelli, Naples, Italy.; ^3^ CEINGE Biotecnologie Avanzate, Naples, Italy.; ^4^ Institute for Applied Mathematics “Mauro Picone,” National Research Council, Naples, Italy.; ^5^ Medical Genetics, Department of Advanced Biomedicine, Federico II University, Naples, Italy.

**Keywords:** dual AAV, kinase inhibitors, retina

## Abstract

Retinal gene therapy based on adeno-associated viral (AAV) vectors is safe and efficient in humans. The low intrinsic DNA transfer capacity of AAV has been expanded by dual vectors where a large expression cassette is split in two halves independently packaged in two AAV vectors. Dual AAV transduction efficiency, however, is greatly reduced compared to that obtained with a single vector. As AAV intracellular trafficking and processing are negatively affected by phosphorylation, this study set to identify kinase inhibitors that can increase dual AAV vector transduction. By high-throughput screening of a kinase inhibitors library, three compounds were identified that increase AAV transduction *in vitro*, one of which has a higher effect on dual than on single AAV vectors. Importantly, the transduction enhancement is exerted on various AAV serotypes and is not transgene dependent. As kinase inhibitors are promiscuous, siRNA-mediated silencing of targeted kinases was performed, and *AURKA* and *B*, *PLK1*, and *PTK2* were among those involved in the increase of AAV transduction levels. The study shows that kinase inhibitor administration reduces AAV serotype 2 (AAV2) capsid phosphorylation and increases the activity of DNA-repair pathways involved in AAV DNA processing. Importantly, the kinase inhibitor PF-00562271 improves dual AAV8 transduction in photoreceptors following sub-retinal delivery in mice. The study identifies kinase inhibitors that increase dual and single AAV transduction by modulating AAV entry and post-entry steps.

## Introduction

Inherited retinal degenerations (IRDs), with an overall global prevalence of 1/2,000,^1^ are a major cause of blindness worldwide. IRDs are mostly monogenic and are caused by mutations in genes mainly expressed in retinal photoreceptors (PR; rods and cones) and to a lesser extent in the retinal pigmented epithelium (RPE).^2^ To date, gene therapy based on adeno-associated viral (AAV) vectors represents the most promising therapeutic approach for IRDs. AAVs are: (1) safe and effective when delivered sub-retinally in patients with Leber's congenital amaurosis type 2, a severe form of inherited childhood blindness^3–8^; and (2) to date, the most effective gene transfer vector for PR in addition to RPE.^9–15^ However, the relatively small DNA packaging capacity of AAV, which is considered to be restricted to the size of the parental genome (4.7 kb),^16–19^ prevents their application from the treatment of those diseases caused by mutations in genes having a coding sequence (CDS) >5 kb. Among these conditions, Stargardt disease (STGD1; MIM_248200) is the most common form of inherited macular degeneration and is caused by mutations in the ATP-binding cassette subfamily A member 4 gene (*ABCA4*; CDS: 6.8 kb),^20^ while Usher syndrome type 1B, a severe form of combined hearing loss and vision loss due to retinitis pigmentosa, is caused by mutations in the gene encoding for Myosin 7A (*MYO7A*; CDS: 6.6 kb).^21^

Dual AAV vectors^22–26^ have been developed to overcome AAV limited cargo capacity by splitting a large transgene expression cassette in two separate halves, each independently packaged in a single normal-size (<5 kb) AAV vector.^22,23,25,27^ The reconstitution of the full-length expression cassette is achieved upon co-infection of both dual AAV vectors in the same cell followed by: (1) inverted terminal repeat (ITR) mediated tail-to-head concatemerization of the 5′ and 3′ genomes and/or homologous recombination between short highly recombinogenic sequences included in both dual AAV vectors^25^; and (2) ITR and recombinogenic sequences splicing out of the mature full-length transgenic transcript.

Dual AAV vectors are particularly suitable for use in the eye, as the small and enclosed sub-retinal environment should favor the co-infection of both dual AAV vectors. Indeed, it was recently shown that dual AAV vectors that contain the short AK recombinogenic region from the F1 phage (dual AAV hybrid vectors) efficiently transduce mouse PR and RPE, and rescue mouse models of IRDs.^26^ In the same study, it was observed that the transduction efficiency of dual AAV hybrid vectors is 6% and 40% of that of a single AAV vector in mice and pigs, respectively. Thus, very high AAV doses are required that pose an increased challenge regarding vector production and the risks of toxicity or vector-directed immune responses, despite the immune-privilege of the sub-retinal space.

Alternatively, the transduction efficiency of dual AAV vectors can be improved by acting on the viral vector infection cycle, which includes viral entry, endosomal trafficking, nuclear entry, second-strand synthesis, and concatemerization/recombination of the viral genomes.^28^ Similar approaches have already been exploited both *in vitro* and *in vivo* to increase AAV transduction efficiency. An example is the co-administration to airway epithelial cells of AAV with calpain inhibitor I, a proteasome inhibitor (PI),^29^ which induces an increase of transduction by inhibiting the proteasome-mediated AAV degradation.^30,31^ In an alternative approach, Nicholson *et al*. performed high-throughput screening (HTS) of Food and Drug Administration (FDA)-approved drugs and identified several compounds that enhance AAV transduction efficiency both *in vitro* and *in vivo* in the mouse liver.^32^

Kinases are known to affect key steps of AAV intracellular trafficking negatively. For example, in HeLa cells, the epidermal growth factor receptor-protein tyrosine kinase (EGFR-PTK) has been reported to act at both the endosomal escape and second-strand synthesis steps, thus negatively modulating AAV transduction efficiency.^33^ Other kinases are thought to affect AAV intracellular trafficking negatively, since AAV vectors with mutated tyrosine, serine, and thereonine residues on the capsid show greater transduction efficiency both *in vitro* and *in vivo*.^34–36^ However, the lack of precise knowledge of the pathways and proteins involved does not allow to define the specific steps of the AAV life cycle that can be modulated to improve AAV transduction.

In this study HTS of the SELLECKCHEM collection of 273 kinase inhibitors was performed in order to identify molecules able to increase the transduction efficiency of dual AAV vectors both *in vitro* and *in vivo* in the mouse retina.

The identification of kinase inhibitors that enhance dual AAV transduction efficiency would both expand the already great applicability of this viral vector platform and allow a better comprehension of the intracellular pathways modulating AAV transduction.

## Methods

### AAV vectors and DNA plasmids

The plasmids used for AAV vector production were derived from pAAV2.1^37^ that contains the ITRs of dual AAV serotype 2 (AAV2). The dual AAV vectors system consists of two separate AAVs: within the ITRs, the 5′ vector carries the promoter, the 5′ coding sequence (CDS), a splicing donor signal (5′-GTAAGTATCAAGGTTACAAGACAGGTTTAAGGAGACCAATAGAAACTGGGCTTGTCGAGACAGAGAAGACTCTTGCGTTTCT-3′) and a recombinogenic sequence derived from the phage F1 genome (AK: J02448.1, bp 5850–5926),^26^ while the 3′ vector plasmid contains the AK sequence, a splicing acceptor signal (5′-GATAGGCACCTATTGGTCTTACTGACATCCACTTTGCCTTTCTCTCCACAG-3′), and the 3′ CDS followed by the simian virus 40 (SV40) polyadenylation signal (pA).

To generate dual AAV vectors for enhanced green fluorescent protein (eGFP) expression, the CDS was split as follow: 5′ = PMID: 9759496, bp 1–393; 3′ = PMID: 9759496, bp 394–720. Either the ubiquitous cytomegalovirus (CMV) or the PR-specific G-protein-coupled receptor kinase 1 (GRK1) promoter were inserted upstream of the 5′ CDS, while the woodchuck hepatitis virus posttranscriptional regulatory element (WPRE) was added between the 3′ CDS and the SV40pA.

In the dual AAV vectors expressing the triple flag (3 × flag) tagged eGFP (eGFP-3 × flag), the CDS of the 3 × flag was cloned at the 3′ terminus of eGFP CDS and the SV40pA was replaced with the bovine growth hormone (bGH) pA sequence.

To generate dual AAV-CMV-ABCA4 vectors, (1) the CDS was split between exons 19 and 20 (5′ half: NM_000350.2, bp 105–3022; 3′ half: NM_000350.2, bp 3023–6926); and (2) the 3 × flag tag CDS was then added at the 3′ terminus of *ABCA4* 3′ CDS.

To generate dual AAV-CBA-MYO7A vectors, (1) the MYO7A CDS was split between exons 24 and 25 (5′ half: NM_000260.3, bp 273–3380; 3′ half: NM_000260.3, bp 3381–6926); and (2) the ubiquitous chicken β-actin (CBA) promoter was inserted upstream of the 5′ CDS, and the 3 × flag tag CDS was added at the 3′ terminus of *MYO7A* 3′ CDS.

The single AAV2 vectors and the DNA plasmids carry a similar expression cassette to that of dual AAV2, except for the presence of an SV40 intron after the CMV promoter and the use of the bGH pA sequence instead of the SV40pA.

### AAV vector production and characterization

AAV vectors were produced by the TIGEM AAV Vector Core using triple transfection of HEK293 cells followed by two rounds of CsCl_2_ purification.^38^ For each viral preparation, physical titers (genome copies [GC]/mL) were determined by averaging the titer achieved by dot-blot analysis^39^ and by polymerase chain reaction (PCR) quantification using TaqMan™ (Applied Biosystems, Carlsbad, CA).^38^ The probes used for dot-blot and PCR analysis were designed to anneal with either the viral promoter or poly-A sequence.

For most of the *in vitro* experiments, AAV2 vectors were used to infect HEK293 cells. In the experiments performed *in vivo* in the mouse retina, AAV8 vectors were used, which efficiently transduce the retinal pigmented epithelium and PRs.^9,10^

### Cell culture

HEK293 cells were maintained in Dulbecco's modified Eagle's medium (DMEM) containing 10% fetal bovine serum, 2 mM of L-glutamine, and 100 × antibiotic-antimycotic (10,000 IU/mL of penicillin, 10,000 μg/mL of streptomycin, and 25 μg/mL of Gibco Amphotericin B; Gibco, Invitrogen S.R.L., Milan, Italy; complete medium).

### HTS

For the automatic screening, made in collaboration with the TIGEM High Content Screening (HCS) Core, cells were plated in 384-well plates at a concentration of 6,000 cell/well in a total volume of 40 μL. The commercially available SELLECK kinase inhibitors library, composed of 273 kinase inhibitors, was kindly provided by the TIGEM HCS core.

Dual AAV2 (5 × 10^4^ GC/cells) and compounds were diluted separately in 5 μL/well of complete medium and then added to the cells.

Before the analysis, cells were fixed with 4% paraformaldehyde in phosphate-buffered saline (PBS) for 7 min and stained with DAPI (1 mg/mL). Images were acquired and analyzed using the OPERA system and the Accapella-based Columbus software, using a script for the evaluation of number of total cells, eGFP-positive cells, and mean fluorescence intensity in eGFP-positive cells.

The screening was performed in triplicate, and Pearson's correlation coefficients were as follows: total number of cells replicate 1 versus 2 = 0.66, replicate 2 versus 3 = 0.71, replicate 1 versus 3 = 0.72; eGFP-positive cells/total cells replicate 1 versus 2 = 0.93, replicate 2 versus 3 = 0.91, replicate 1 versus 3 = 0.89; mean fluorescence intensity in eGFP-positive cells replicate 1 versus 2 = 0.86, replicate 2 versus 3 = 0.89, replicate 1 versus 3 = 0.83. Pearson's correlation values support the reproducibility of the screening replicates.^40^

### AAV infection and transfection

HEK293 cells were plated at a concentration of 5 × 10^5^ cell/mL (with the exception of experiments in Figs. 2 and 3 where cells were plated at 1 × 10^6^ cell/mL). Hela cells were plated at a concentration of 1.5 × 10^5^ cell/mL.

For Western blot, RNA, and cytofluorimetric analysis, 2 mL/well (or 500 μL/well) of cells were plated in six-well plates (or 24-well pates). For AAV infection, 24 h later, cells were washed once with PBS and then infected with the indicated virus (multiplicity of infection [MOI]: single AAV2-eGFP = 2.5 × 10^3^ GC/cell; dual AAV2 = 5 × 10^4^ GC/cell of each vector; dual AAV1, 5, 8, 9, rh10 = 1 × 10^5^ GC/cell of each vector) in a final volume of 700 μL/well (or 250 μL/well) of serum-free DMEM. Two hours post infection, every well received 1.3 mL (or 250 μL/well) of fresh, preheated, complete DMEM supplemented with the drugs at the desired concentrations.

Plasmid and siRNA (Ambion Thermo Fisher Scientific, Waltham, MA) transfection was performed in six-well plates with the calcium phosphate method. Twenty-four hours after seeding (or at the time of seeding for siRNA), plasmid or siRNA of interest was diluted in 150 μL/well of CalCl_2_, and 150 μL/well of HEPES-buffered saline 2 × was added to the mix. After 3 min of incubation, the suspension was added to the cells. For plasmid transfection, media was replaced 4 h later with complete DMEM supplemented with the drugs at the desired concentrations. For siRNA-mediated knockdown, media was not replaced, and cells were harvested 24 h after transfection.

For the immunoprecipitation experiment, 20 mL of HEK293 cells were plated in 15 cm dishes. Twenty-four hours later, cells were pretreated with the drugs for 1 h (F13 = 5 μM; H17 = 10 μM; K17 = 1 μM; dimethyl sulfoxide [DMSO], the drug solvent, at the maximum volume used to dissolve the drugs), washed once with PBS, infected with AAV2 vectors (MOI = 1 × 10^5^) in 10 mL of serum-free DMEM, and harvested 5 min later.

For the study of the activation of ataxia-telangiectasia mutated (ATM) pathway, 20 mL of HEK293 cells were plated in 15 cm dishes. Twenty-four hours later, cells were infected with AAV2 vectors (MOI = 1 × 10^5^) in 10 mL of serum-free DMEM. Two hours post infection, cells received 10 mL of fresh, preheated, complete DMEM supplemented with the kinase inhibitors (F13 = 5 μM; H17 = 10 μM; K17 = 1 μM; DMSO, the drug solvent, at the maximum volume used to dissolve the drugs) and were harvested 1 h later for analysis.

### RNA extraction, cDNA production, and reverse transcription analysis

Total RNA was extracted using the reverse transcription (RT)-PCR RNeasy MiniKit (Qiagen, Milan, Italy). RNA (250–500 ng) was submitted to DNase I digestion (RNase Free DNase set; Qiagen), and cDNA was generated using the QuantiTect reverse transcription kit (Qiagen). For each sample, the same amount of RNA did not receive the retrotranscriptase enzyme and was used as a control for genomic DNA contamination.

Primers for RT-qPCR were purchased either from Sigma–Aldrich (Milan, Italy) or Eurofins (Milan, Italy; sequence available upon request). The SybrGreen RT-qPCR kit was purchased from Roche (Monza, Italy) and performed following the manufacturer's protocol on the LyghtCycler™ 96 system (Roche). Diluted cDNA (5 μL of 1:10 for gene knock down experiment; 1:50 for transgene RNA quantification) and 10 pmol of primers in a total volume of 20 μL were used for PCR. Thermal cycling for all genes initiated with a denaturation step at 95°C for 5 min, followed by 45 cycles with denaturation at 95°C for 10 s, annealing at 60°C for 20 s, and extension at 72°C for 20 s.

To calculate the mRNA expression levels, the ΔΔCq formula was used:

\begin{align*}
\Delta \Delta { \rm{Cq}} \ =  \left[ {{2^{ - ( { \rm{Cqsample  -
Cqsample - actin}} ) }}} \right] /
\\ \quad\quad\quad\quad \left[ {{2^{ - ( { \rm{Cqscramble  - Cqscramble - actin}} ) }}} \right].
\end{align*}

Standard curves for determining the efficiency values were calculated by the machine software and were generated by diluting cDNAs 1:5–1:50–1:500–1:5,000–1:50,000 in case of highly represented transcripts or 1:1–1:10–1:100–1:1,000–1:10,000 for poorly represented transcripts; only efficiency values ranging from 1.80 to 2.20 were considered acceptable.

### Cytofluorimetric analysis

Seventy-two hours post infection, HEK293 cells were washed with PBS, harvested by tripsinization, washed with PBS 5 mM EDTA, re-suspended in 400 μL of PBS 5 mM EDTA, and analyzed with a DB Accury™ C6 flow cytometer system (DB Biosciences, San Jose, CA).

As the presence of the compounds might change the background autofluorescence of the cells, for each sample of infected cells, those that were not infected and were incubated with the corresponding compound were considered as blank. The threshold for considering the eGFP fluorescence as positive was placed at a value where the negative control showed 1% of positive events. For calculating the fluorescence, the total fluorescence of positive cells was divided by the total fluorescence of negative cells.

### Analysis of immunoprecipitates

Five minutes post infection, HEK293 cells were harvested and lysed (at 1.5 mg/mL protein concentration) as described,^41^ using a lysis buffer containing 50 mM of HEPES, pH 7.5, plus 150 mM of NaCl, sodium deoxycholate 0.25%, Nonidet P40 1%, 1 mM of EDTA, 25 mM of beta-glycerophosphate, 10 μg/mL of aprotinin, 50 mM of NaF, 1 mM of sodium orthovanadate, 1 mM of PMSF, and a cocktail of protease inhibitors (leupeptin, antipain, pepstatin; LAP; Sigma–Aldrich, St. Louis, MO), which was used according to the manufacturer's instructions. AAV VP capsid proteins were immunoprecipitated using 1 μg of anti-VP mouse monoclonal antibody (Clone B1; Progen, Heidelberg, Germany). Mouse monoclonal anti-P-tyrosine antibody (1:1,000, clone 4G10; Millipore, Darmstadt, Germany) was used to detect P-Tyr phosphorylated proteins. Mouse monoclonal anti-P-serine antibody (1:500, clone16B4, cat. sc-81514; Santa Cruz Biotechnology, Dallas, TX) was used to detect P-Ser phosphorylated proteins. Integrin beta1 was revealed using rabbit polyclonal anti-integrin beta1 antibody (1:1,000, AB1952; Millipore).

Sodium dodecyl sulfate polyacrylamide gel electrophoresis (SDS-PAGE) and Western blot were performed as described.^41^ Immunoreactive proteins were revealed using the enhanced chemilumescent detection system (GE Healthcare, Chicago, Illinois).

### Enzyme-linked immunosorbent assay for human phospho-ATM (Ser1981) detection

One hour post compounds addition, HEK293 cells were harvested with PBS and were then lysed in 250 μL of lysis buffer provided in the Human Phospho-ATM (S1981) enzyme-linked immunosorbent assay (ELISA) kit (Ray Biotech, Inc., Norcross, GA) supplemented with phosphatase (cat. P5726 and p0044; Sigma–Aldrich) and protease (Complete Protease inhibitor cocktail tablets; Roche) inhibitors. The ELISA was then performed following the manufacturer instruction using 100 μg of protein/sample. The optical density (OD) at 450 nm was read on a Promega GloMax^®^-Multi Detection System (Promega, Madison, WI). Results are presented in a percentage of the OD 450 measured in uninfected cells incubated with DMSO.

### Subretinal injection of AAV vectors in mice

This study was carried out in accordance with the Association for Research in Vision and Ophthalmology Statement for the Use of Animals in Ophthalmic and Vision Research and with the Italian Ministry of Health regulation for animal procedures. All procedures on mice were submitted to the Italian Ministry of Health, Department of Public Health, Animal Health, Nutrition, and Food Safety on October 17, 2011. The Ministry of Health approved the procedures by silence/consent, as per article 7 of the 116/92 Ministerial Decree. Four-week-old C57BL/6 mice were purchased from Envigo RMS SRL (Udine, Italy) and were housed at the TIGEM animal house (Naples, Italy) and maintained under a 12 h light/12 h dark cycle (10–50 lux exposure during the light phase). Surgery was performed under anesthesia, and all efforts were made to minimize animal suffering. Mice (4–5 weeks old) were anesthetized with an intraperitoneal injection of 0.3 mL of ketamine +1:2 xilazin, diluted 1:10 in saline solution (final concentration 1.5 mL/100 g body weight); mice were sub-retinally injected via a trans-scleral choroidal approach, as described by Liang *et al.*^42^ with 1 μL of a mix containing AAV8 vector solution and one drug (initially prepared in DMSO) at the indicated concentrations (10, 30, and 100 μM). DMSO control animals received the same AAV8 vector mix with a 0.1% final concentration of DMSO (the maximum drug percentage present in the mix of treated animals). Two months post injection, mice were sacrificed by cervical dislocation, and eyecups (cups + retinas) or retinas alone were harvested and analyzed by Western blot.

### Western blot analysis

For Western blot analysis, HEK293 cells, eyecups, or neural retinas were lysed in the radioimmunoprecipitation assay (RIPA) buffer (50 mM of Tris-HCl, pH 8.0, 150 mM of NaCl, 1% NP40, 0.5% Na-Deoxycholate, 1 mM of EDTA, 0.1% SDS, pH 8.0) supplemented with protease inhibitors (Complete Protease inhibitor cocktail tablets; Roche) and 1 mM phenylmethanesulfonyl fluoride. After 30 min of lysis and one cycle of freeze and thaw, every sample was submitted to the Pierce BCA protein assay kit (Thermo Fisher Scientific) to measure the protein concentration in the lysate. Samples (50 μg for cells and between 45 and 100 μg for eyecups and neural retinas) were denatured at 99°C for 5 min in 4 × sample dye (SDS powder, 100% glycerol, β-mercapthoethanol, Tris pH 6.8 1M, Blu Bromophenol) and separated by 12% SDS-PAGE. The antibodies used for immunoblotting are as follows: anti-eGFP (1:1,000, cat. sc-8334; Santa Cruz Biotechnology); anti-3xflag (1:1,000, cat. A8592; Sigma–Aldrich); anti-β-tubulin (1:1,000, cat. A8592; Sigma–Aldrich); anti-β-actin (1:1,000, cat. NB600-501; Novus Biological LLC, Littleton, CO); anti-Calnexin (1:1,000, cat. ADI-SPA 860; Enzo Life Sciences, Farmingdale, NY). eGFP band intensity was measured from the Chemidoc (Amersham Imager 600; GE Healthcare) pictures using ImageJ software and normalized over β-tubulin or β-actin bands in the *in vitro* experiments, and over Calnexin bands in the *in vivo* experiment involving dual AAV8-GRK1-eGFP. In the *in vivo* experiment involving dual AAV8-CMV-eGFP, the same amount of protein lysate (100 μg) was loaded for all samples.

In order to quantify eGFP bands from the Western blot of retinal lysates and to allow comparison between samples in different blots, in each gel, a standard lysate of transfected HEK293 cell was loaded. For the Western blot with anti-eGFP antibodies, 0.5 μg lysate of cells transfected with 1 μg CMV-eGFP plasmid was used, while for Western blot with anti-3xflag antibodies (Fig. 7), 20 μg lysate of cells transfected with 10 ng CMV-eGFP-3xflag plasmid was used.

### Electrophysiological recordings

For electroretinographic analyses, mice were first dark-adapted for 3 h and then anesthetized and positioned in a stereotaxic apparatus under a dim red light. Their pupils were dilated with a drop of 0.5% tropicamide (Visufarma, Rome, Italy), and their body temperature was maintained at 37.5°C. Light flashes were generated by a Ganzfeld stimulator (CSO; Costruzione Strumenti Oftalmici, Florence, Italy). The electrophysiological signals were recorded through gold-plate electrodes inserted under the lower eyelids in contact with the cornea. The electrodes in each eye were referred to a needle electrode inserted subcutaneously at the level of the corresponding frontal region. The different electrodes were connected to a two-channel amplifier. After completion of responses obtained in dark-adapted conditions (scotopic), the recording session continued with the purpose of dissecting the cone pathway mediating the light response (photopic). To minimize the noise, different responses evoked by light were averaged for each luminance step. The maximal scotopic response of rods and cones was measured in dark conditions (scotopic) with two flashes of 0.7 Hz and a light intensity of 20 cd s/m^2^. Photopic cone responses were isolated in light conditions with a continuous background white light of 50 cd s/m^2^, with 10 flashes of 0.7 Hz and a light intensity of 20 cd s/m^2^.

### Spectral domain optical coherence tomography

Spectral domain optical coherence tomogram images were obtained using the Bioptigen Spectral Domain Ophthalmic Imaging System (SDOIS; Envisu R2200; Bioptigen, Inc., Morrisville, NC). Mice were anesthetized, and pupils were dilated by applying one or two drops of topical 0.5% tropicamide (Visufarma, Rome, Italy). To prevent corneal desiccation during the procedure, topical lubricant eye drops (Recugel; Bausch & Lomb, Rochester, NY) were applied bilaterally with a small brush. Mice were positioned into the animal imaging mount and rodent alignment stage (AIM-RAS; Bioptigen, Inc.). The laser source was placed in front of the mouse, and images were acquired by the InVivoVue Clinic software (Bioptigen, Inc.). Three images—one central, one superior, and one inferior to the optic nerve—were taken from each eye. Outer nuclear layer thickness was manually measured three times from each OCT scan image and averaged.

### Statistical analysis

Statistical *p*-values ≤0.05 were considered significant. For the RNA, cytofluorimetric and ELISA analysis, a one-way analysis of variance (ANOVA) was performed, followed by Dunnett's *post hoc* test. For the eGFP RNA analysis, a *p*-value ANOVA of 2.75 × 10^–4^ and *p*-values for F13, H17, and K17 versus DMSO of <0.0001 were measured. In Fig. 4A, a *p*-value ANOVA of 1.91 × 10^–7^ and *p-*values for F13, H17, and K17 versus DMSO of <0.0001 were measured. In Fig. 4B, a *p*-value ANOVA of 4.9 × 10^–5^ and *p*-values versus DMSO of F13 = 0.0058, H17 < 0.0001, and K17 = 0.0001 were measured. In Fig. 4C, the following were statistically significant differences versus DMSO: AAV1 *p*-value ANOVA = 3.62 × 10^–5^, F13 < 0.001, H17 = 0.004, K17 = 0.019; AAV8 *p*-value ANOVA = 0.010, F13 = 0.005, H17 = 0.044; AAV9 *p*-value ANOVA = 0.001, F13 = 0.006, H17 < 0.001; and AAVrh10 *p*-value ANOVA = 0.006, H17 = 0.003. In Fig. 6B, the following were statistically significant differences: *p*-value ANOVA = 0.001, and AAV + H17 versus AAV + DMSO = 0.005. In Supplementary Fig. S1 (Supplementary Data are available online at www.liebertpub.com/hum), the following were statistically significant differences: *p*-value ANOVA = 7.02 × 10^–7^, F13 + K17 versus F13 = 0.002, F13 + K17 versus K17 = 0.001, H17 + K17 versus H17 < 0.001, and H17 + K17 versus K17 < 0.001.

To test whether the effects of the compounds might be synergistic, the values obtained in samples incubated with the compounds were summed separately, and this was compared to the values measured in cells incubated with the combination of the corresponding compounds using a one-tailed Student's *t*-test. The following were statistically significant differences: F13 + K17 = 0.054 and H17 + K17 = 0.048.

To compare the enhancement of protein expression to the DMSO control (evaluated by Western blot), the one-tailed nonparametric Mann–Whitney–Wilcoxon test was used, where *p*-values were corrected for multiplicity using the Benjamini–Hochberg procedure.^43^ For the eGFP enhancement measured with dual AAV2, the data from Fig. 3 were used, and the following were statistically significant differences versus DMSO: PI 7.5 μM = 9.5 × 10^–6^; B19 10 μM = 0.035; F13 5 μM = 0.005; F19 10 μM = 0.013; H17 3 μM = 0.035; H17 10 μM = 0.035; K17 0.3 μM = 0.035; K17 1 μM = 0.035; N9 10 μM = 0.035. In Fig. 4A, all the samples were statistically significant versus the DMSO, with *p-*values of 0.008. In Fig. 4B, all samples were statistically significant versus DMSO, with the following *p-*values: F13 = 0.013, H17 = 0.019, and K17 = 0.019. Please note that in Fig. 4A and B, in each panel, two independent analyses were performed for the RNA and for the proteins. In Fig. 5, the following were statistically significant differences versus the scramble: siAURKA = 0.026, siAURKB = 0.039, siPTK2 = 0.032, and siPLK1 = 0.026. In Fig. 7B, the following were statistically significant differences versus DMSO: PI 7.5 μM = 0.027 and F13 30 μM = 0.017. In Fig. 3, to compare the enhancement in protein expression between different systems, the nonparametric Kruskall–Wallis test was used, with Dunn's multiple comparison test as a *post hoc* procedure. The following were statistically significant differences. PI 7.5 μM group: dual AAV versus plasmid = 0.000055 and single AAV versus plasmid = 0.000044. F13 5 μM group: dual AAV versus plasmid = 0.0024 and single AAV versus plasmid = 0.0113. F19 10 μM group: single AAV versus plasmid = 0.0053. H17 10 μM group: dual AAV versus plasmid = 0.024 and single AAV versus plasmid = 0.016. K17 0.3 μM group: dual AAV versus plasmid = 0.0021. K17 1 μM group: dual AAV versus plasmid = 0.0073. In Supplementary Fig. S2, a statistical modeling approach was performed based on Gaussian processes, as described by Kalaitzis *et al.*,^44^ and no significant differences were found for the different treatments.

## Results

### HTS identifies seven compounds that enhance dual AAV transduction

To identify compounds able to increase the transduction efficiency of dual AAV vectors, HTS of a kinase inhibitors library (SELLECKCHEM) was performed in triplicate on HEK293 cells infected with dual AAV2 vectors encoding for the reporter eGFP.

**Figure 1.**
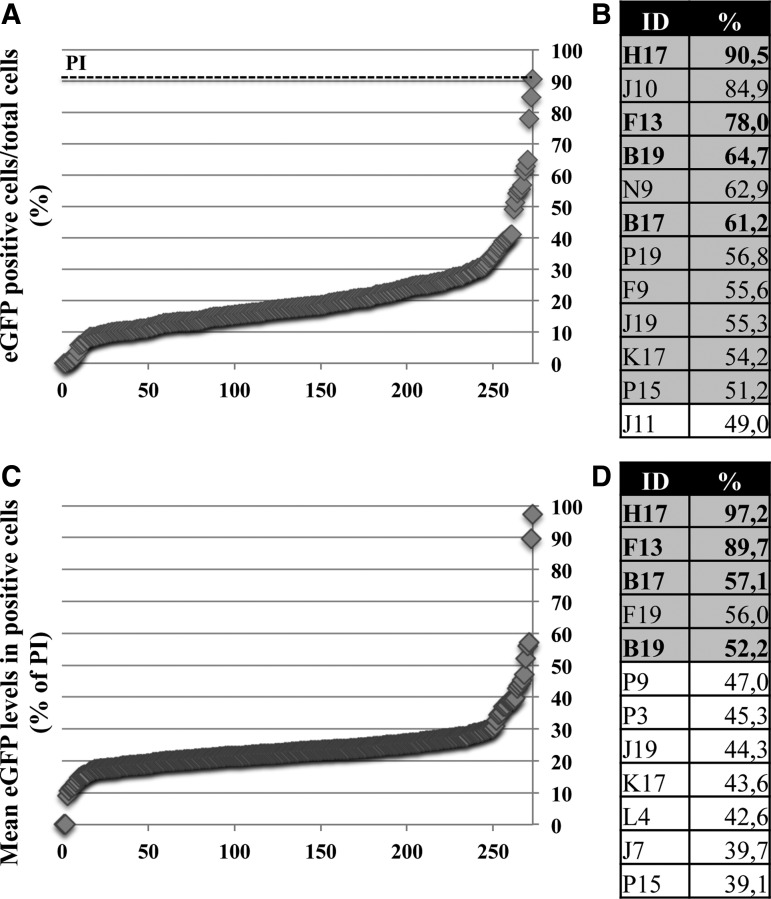
High-throughput screening of 273 kinase inhibitors identifies 12 compounds that enhance adeno-associated viral (AAV) transduction. **(A** and **B)** Enhanced green fluorescent protein (eGFP)-positive cells/total cells: results from the analysis were organized and plotted in ascending value both in the chart **(A)** and in the table **(B)**. The *dashed line* indicates the value obtained with the PI as positive control. **(C** and **D)** Mean fluorescence intensity in eGFP-positive cells normalized to the mean eGFP fluorescence intensity measured in the positive control (PI 7.5 μM): results from the analysis were organized and plotted in ascending value both in the chart **(C)** and in the table **(D)**. PI, proteasome inhibitor (Calpain inhibitor 1); ID, identification number (according to the coordinates on the 384-well plate). Values are presented as the mean of three independent experiments.

Twenty-four hours after seeding, cells were administered with both dual AAV2 vectors expressing eGFP under the control of the ubiquitous CMV promoter (MOI = 5 × 10^4^ GC/cell of each vector) and each of the 273 Selleck compounds at a final concentration of 10 μM. Seventy-two hours after incubation, cells were fixed and analyzed (Fig. 1) using the high-content and high-throughput OPERA system to acquire images and a dedicated script using the Acapella-based Columbus software (Perkin Elmer, Waltham, MA) for analysis. Untreated cells served as negative control of infection. Infected cells incubated with the drug solvent (DMSO) served as baseline reference. Infected cells incubated with 7.5 μM of the PI Calpain inhibitor I served as a positive control.^29^ The PI optimal dosage was established after a dose–response study (data not shown). Positive hits were selected through a custom-made script, taking into account: (1) percentage of eGFP-positive cells/total cells (Fig. 1A and B), where PI treatment resulted in 91% of eGFP-positive cells/total cells, and (2) mean eGFP fluorescence intensity in eGFP-positive cells (percentage of PI; Fig. 1C and D). Compounds able to achieve a score >50% for one or both parameters were arbitrarily rated as positive. Using such a threshold, 12 compounds were selected (Fig. 1B and D), four of which (H17, F13, B17, and B19, in bold; the nomenclature refers to the coordinates on the 384-well plate) were positive for both parameters. The reproducibility of the results from three independent experiments is supported by the Pearson's correlation factor values (see Methods). Next, to determine the optimal concentration of each compound, a drug–dose response was performed, with concentrations ranging from 0.1 to 30 μM. Optimal dosages were obtained by balancing the eGFP intensity enhancement with toxicity measured as number of cells surviving after the treatment (Supplementary Fig. S3). Specifically, the following concentrations were selected: B17 3 μM, B19 3 μM, F9 1 μM, F13 5 μM, F19 10 μM, H17 3 and 10 μM, J10 30 μM, J19 10 μM, K17 0.3 and 1 μM, P15 10 μM, P19 10 μM, and N9 10 μM.

**Figure 2.**
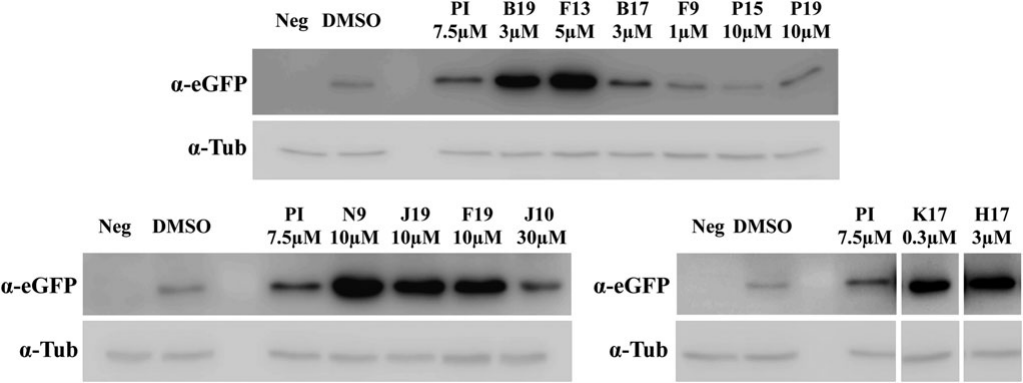
Seven kinase inhibitors enhance dual AAV serotype 2 (AAV2) transduction *in vitro* at higher levels than PI. Representative Western blot (WB) analysis (*n* ≥ 2 replicates) of lysates from HEK293 cells infected with dual AAV2 vectors expressing eGFP and incubated with the drugs at the indicated concentrations. For each sample, 50 μg of protein were loaded. α-eGFP, WB with anti-eGFP antibody; α-tub, WB with anti-tubulin antibody, used as loading control; Neg, not treated cells; dual AAV, dual AAV2 expressing eGFP; DMSO, dimethyl sulfoxide (baseline control, the drug solvent).

To validate the increased levels of eGFP expression observed in the 384-well plate format, HEK293 cells were seeded in six-well plates at *t* = 0 and infected at *t* = 24 h with dual AAV2 vectors (MOI = 5 × 10^4^ GC/cell of each vector). The drugs were added to the cells at the optimal concentration 2 h post infection (*t* = 26 h). At *t* = 96 h, cells were harvested, lysed, and analyzed by Western blot with anti-eGFP antibodies (Fig. 2). Seven (B19, F13, F19, K17, H17, J19, and N9) out of 12 compounds resulted in an increment of eGFP expression higher than that observed with PI 7.5 μM (used as a positive control). This increment was statistically significant for all drugs except for J19.

### Selective induction of dual and single AAV transduction by some of the compounds identified by the screening

The increment in eGFP expression observed upon incubation with the selected drugs could be the result of effects on single or dual-specific AAV-mediated transduction or eGFP transgene expression independent of AAV infection or transduction.

**Figure 3.**
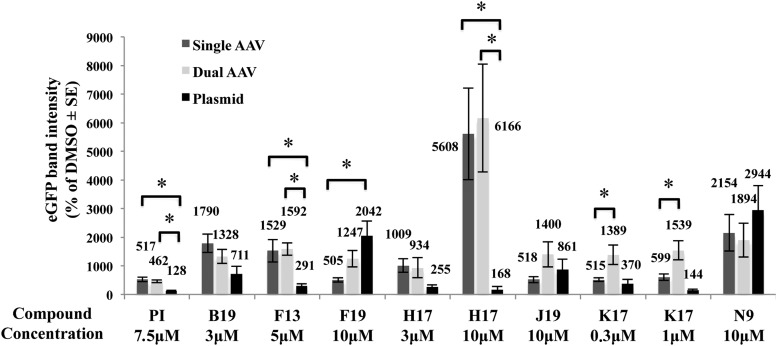
Selective enhancement of drug-induced transduction. eGFP protein quantification by WB (*n* ≥ 5 replicates) of lysates from HEK293 cells infected with either single (*medium-gray bars*) or dual AAV2 (*light-gray bars*) vectors or transfected with a plasmid expressing eGFP (*dark-gray bars*), and incubated or not with the drugs at the indicated concentrations. Values, normalized to the DMSO sample present in every WB, are presented as the mean ± standard error of the mean (SE). *p < 0.05.

To investigate this, the increment of eGFP expression upon the addition of compounds to HEK293 cells either transfected with an eGFP-expressing plasmid (100 ng/well of a six-well plate) or infected with single (MOI = 2.5 × 10^3^ GC/cell) or dual AAV2 vectors (MOI = 5 × 10^4^ GC/cell of each vector) was measured. Both the amount of transfected plasmid and the MOI of the single AAV vector used were selected to result in similar levels of baseline transgene expression, which was below the levels that saturate cell expression machinery to allow for further kinase inhibitor induction. To allow transgene expression to occur, the various compounds were added either 4 h post transfection or 2 h post infection. Notably, K17 and H17 were tested at two different concentrations: the higher concentration served to obtain the maximum induction, while the lower served to maintain the toxicity at an acceptable level at the cost of a lower induction. At *t* = 96 h, cells were harvested, lysed, and analyzed by Western blot with anti-eGFP antibodies (Fig. 3). eGFP expression was normalized to the corresponding untreated sample (DMSO). After statistical analysis of the results, compounds can be classified in three groups based on their effects on eGFP expression in the three systems: (1) compounds that do not significantly increase eGFP expression between the three systems: B19 3 μM, F19 10 μM, J19 10 μM, and N9 10 μM; (2) compounds that significantly increase eGFP expression between AAV (whether single or dual) and plasmid: PI 7.5 μM, F13 5 μM, and H17 10 μM (H17 3 μM shows a tendency but was not statistically significant); and (3) compounds that significantly increase eGFP expression between single and dual AAV: K17 at 1 μM and 0.3 μM. Kinase inhibitors belonging to group (1) were discarded, as their effect was not considered to be AAV specific.

To understand if the increment of eGFP expression mediated by F13, H17, and K17 occurs at the transcriptional rather than post-translational level, eGFP RNA levels were measured, and these were 609% (F13 5 μM), 746% (with H17 3 μM), and 1,445% (with K17 0.3 μM) of those measured in infected cells incubated with DMSO.

**Figure 4.**
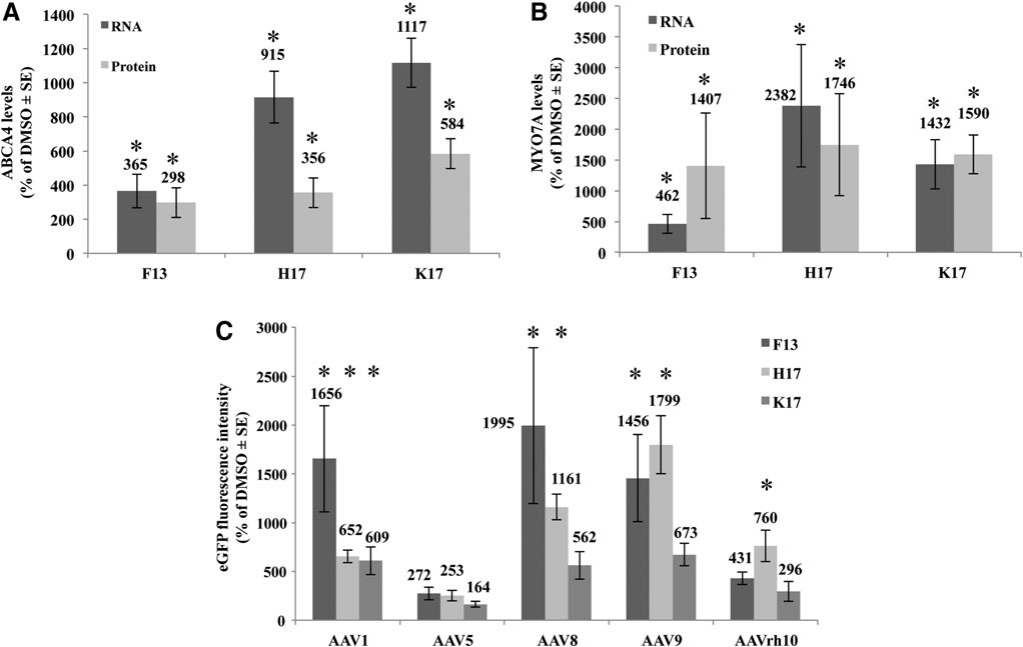
The kinase inhibitors enhancement of dual AAV transduction occurs with different transgenes and AAV serotypes. **(A** and **B)** RNA (*dark gray*; *n* = 4) and protein (*light gray*; *n* ≥ 4) quantification measured by reverse transcription quantitative polymerase chain reaction and WB analysis, respectively, in lysates from HEK293 cells infected with dual AAV2 vectors expressing either ABCA4 **(A)** or MYO7A **(B)** and incubated with the various drugs. **(C)** Cytofluorimetric analysis (*n* = 3) of eGFP expression in HEK293 cells infected with various dual AAV-eGFP serotypes and incubated with F13 5 μM (*dark gray*), H17 3 μM (*light gray*), and K17 0.3 μM (*medium gray*). Values, normalized to the corresponding DMSO sample, are presented as the mean ± SE. *p < 0.05.

To evaluate whether the transduction enhancement observed with F13, H17, and K17 is independent of the transgene expressed, HEK293 cells were infected with dual AAV2 that express either *ABCA4* (6.8 kb) or *MYO7A* (6.7 kb), which are causative of STGD1, the most common form of inherited macular degeneration, or USH1B, one of the most severe forms of inherited combined RP and deafness, respectively. The infection was carried out using conditions similar to those used with dual AAV2 vectors that express eGFP (MOI = 5 × 10^4^ GC/cell of each vector; infection *t* = 24 h; compound addition *t* = 26 h; harvesting *t* = 96 h). RNA and protein quantification performed by RT-qPCR and Western blot analysis, respectively, confirmed that all three molecules induce a significant increment in both ABCA4 (Fig. 4A) and MYO7A (Fig. 4B) expression compared to the corresponding untreated samples (DMSO). While the level of transcriptional enhancement was comparable for the three transgenes when cells were treated with the same compound, an overall higher increment of protein levels was observed for eGFP and MYO7A than for ABCA4 (Figs. 3 and 4A and B).

To evaluate whether the effect of the three kinase inhibitors is AAV serotype-dependent, dual AAV-EGFP vectors of the most used AAV serotypes belonging to various AAV clades were generated: AAV1 for clade A, AAV8 and AAVrh10 for clade E, AAV9 for clade F, and AAV5, which is very divergent from other AAV serotypes.^45^ The AAV2 used in this study belongs to clade B.^45^ The infection protocol was similar to the one used for the Western blot analysis (except for the MOI of 1 × 10^5^ GC/cell of each vector). The eGFP expression levels were evaluated by cytofluorimetric analysis.

As shown in Fig. 4C, eGFP expression levels mediated by AAV1 were significantly increased by the addition of each of the three compounds, while both F13 and H17 increased AAV8 and AAV9 expression, and only H17 induced a significant increase in eGFP expression from AAVrh10. No significant drug-induced increments of eGFP expression were observed with AAV5. Importantly, the eGFP fluorescence measured in cells infected with dual AAV5 and rh10 and incubated with DMSO as baseline control was the lowest among the serotypes tested (% of EGFP-positive cells: AAV1 = 2.73; AAV5 = 0.07; AAV8 = 0.10; AAV9 = 0.12; AAVrh10 = 0.08), which indicates a particularly low ability by these serotypes to transduce HEK293 cells.

To test whether the effects of the three compounds might be additive, synergistic, or detrimental, HEK293 cells were infected (MOI = 5 × 10^4^ GC/cell of each vector; infection *t* = 24 h; compound addition *t* = 26 h; harvesting *t* = 96 h) with dual AAV2 vectors expressing eGFP, and they were incubated with all possible combinations of compounds (F13 5 μM, H17 3 μM, and K17 0.3 μM). eGFP expression was then evaluated by cytofluorimetric analysis. While the samples incubated with F13 + H17 and F13 + H17 + K17 showed a decrease in eGFP expression when compared to the samples incubated with single drugs, the samples incubated with F13 + K17 and H17 + K17 showed a strong increase in the reporter protein expression, suggesting a synergistic effect of the two sets of drugs (Supplementary Fig. S1).

### Kinase knockdown increases AAV transduction

To confirm which of the kinases known to be inhibited by F13, K17, and H17 (Table 1) specifically impacts on dual AAV-mediated transduction, they were knocked down singularly using siRNA, and dual AAV2 transduction of HEK293 cells was evaluated. HEK293 cells were transiently transfected with siRNAs, including a scramble siRNA as negative control. First, kinase silencing was evaluated by RT-qPCR at *t* = 24 h, the time of dual AAV2 infection. Residual mRNA levels ranging between 14.03% and 26.70% were observed (Supplementary Fig. S4) for all targets. mRNA levels of *NTRK1* could not be evaluated due to its very low/undetectable baseline expression level.

**Table 1.** Positive hits and their kinase targets

*Hits*	*SELLECK compound*	*Main targets*
F13	PF-00562271	***PTK2***^54^
H17	PF-03814735	*AURKA*, ***AURKB***, ***FLT3***, *NTRK1*, ***PTK2***^55^
K17	BI2536	*PLK1*, *PI3K*^56^
B19	Crenolanib (CP-868596)	*PDGFRα*, *PDGFRβ*,^57^***FLT3*** (www.selleckchem.com)
F19	Fedratinib (TG101348, SAR302503)	*JAK2*^58^
J19	GSK1070916	***AURKB***, *AURKC*^59^
N9	Fostamatinib (R788)	*SYK*^60^

Columns from the left report: the identification number (ID) of the compounds based on their position within the 384-well plate, the name of the compounds, and their main kinase targets. Compounds shown to specifically increase adeno-associated virus (AAV) transduction are F13, H17, and K17. Targets that are inhibited by more than one compound are highlighted in bold.

**Figure 5.**
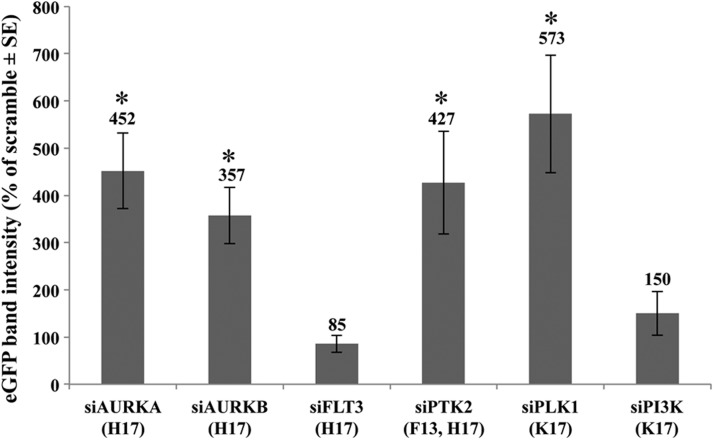
Kinase knockdown improves dual AAV2 transduction efficiency *in vitro*. eGFP protein quantification by WB (*n* ≥ 5) of lysates from HEK293 cells transfected with 50 nM of the indicated siRNA and infected with dual AAV2 vectors expressing eGFP. Values, normalized to the scramble sample present in every WB, are presented as the mean ± SE. The SELLECK compound specific for the given kinase are indicated in parentheses. *p < 0.05.

Twenty-four hours after transfection with siRNAs, HEK293 cells were infected with dual AAV2 vectors expressing eGFP (MOI = 5 × 10^4^ GC/cell of each vector). At *t* = 96 h, cells were harvested and lysed for Western blot analysis with an anti-eGFP antibody, which showed that silencing of *AURKA*, *AURKB*, *PTK2*, and *PLK1* results in a significant increase of eGFP expression (Fig. 5).

### Kinase inhibitors enhance AAV2 capsid interaction with β1-integrin, decrease capsid phosphorylation, and activate ATM

To shed light on the mechanism by which kinase inhibitors increase AAV transduction, the following were evaluated: AAV2 capsid interaction with α5β1 integrin, which is an AAV co-receptor in HEK293 cells^46^; AAV2 capsid phosphorylation, which has been described to target AAV particles to proteasome degradation^29^; and ATM activation, which favors AAV genome circularization.^47^

**Figure 6.**
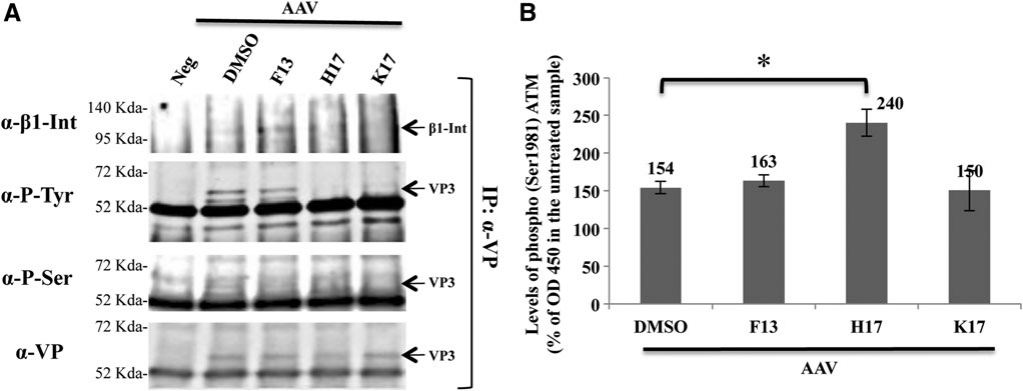
Kinase inhibitors effects on AAV2 entry, capsid phosphorylation, and ataxia-telangiectasia mutated (ATM) activation. **(A)** Representative WB analysis (*n* = 2) of immunoprecipitates (IP) from lysates of HEK293 cells pretreated with the various drugs and infected with dual AAV2 vectors expressing eGFP. Immunoprecipitates were blotted with anti-β1 integrin (α-β1-Int), anti-phospho-tyrosine (α-P-Tyr), or anti-phospho-serine (α-P-Ser) antibodies indicated on the *left*. Parallel samples of the same immunoprecipitates were blotted using the anti-VP (α-VP) antibodies. Molecular weight markers are shown on the *left*. IP, immunoprecipitation; arrows point at β1 integrin and AAV VP3. **(B)** Phospho (Ser 1981) ATM measured by enzyme-linked immunosorbent assay in lysates of HEK293 cells infected for 2 h with dual AAV2 and incubated for 1 h with the kinase inhibitors. Values (*n* = 3) are expressed as the mean percentage of the values measured in cells not infected and incubated with DMSO ± SE. Drug concentrations: F13 5 μM, H17 10 μM, and K17 1 μM. *p < 0.05.

HEK293 cells were incubated with F13 5 μM, H17 10 μM, K17 1 μM, or DMSO, as negative control, 1 h before being infected with dual AAV2 vectors (MOI = 1 × 10^5^ GC/cell). Cells were harvested 5 min after infection, the time point at which the maximal AAV capsid phosphorylation within infected cells was observed (data not shown). HEK293 cell lysates were immunoprecipitated with anti-VP antibodies followed by Western blot with anti-phosphotyrosine, -phosphoserine, -β1 integrin, or -VP antibodies (Fig. 6A). It was confirmed that AAV VP proteins associate with β1 integrin and that this association is increased by the PTK2 inhibitor F13 (Fig. 6A). In addition, it was observed that both H17 and K17, and to a lesser extent F13, reduce tyrosine and serine phosphorylation of VP3, the most abundant VP protein within AAV capsids,^48^ which could unequivocally be identified above background (Fig. 6A). Finally, the study tested whether a 1 h treatment with either F13 5 μM, H17 10 μM, or K17 1 μM promotes ATM activation in HEK293 cells infected 2 h before with dual AAV2 vectors (MOI = 1 × 10^5^ GC/cell). Cells lysates were analyzed by ELISA for the quantification of ATM phospho Ser1981 as a marker of ATM activation. Administration of H17, AAV2, or both increases ATM activation (Fig. 6B).

### F13 (PF-00562271) improves dual AAV8 transduction efficiency in mouse PRs

As the retina is a desirable target of AAV-mediated gene therapy, the ability of kinase inhibitors to enhance dual AAV-mediated expression *in vivo* was evaluated. F13, H17, and K17 were selected based on their robust induction of AAV-mediated expression *in vitro* and their selectivity for AAV transduction. For retinal transduction, recombinant vectors based on AAV8 vectors were used, which transduce both retinal pigmented epithelium and PR more efficiently than AAV2 used for the *in vitro* experiments.^9,10^ Dual AAV8 vectors expressing eGFP under the control of the ubiquitous CMV promoter (dose 4.1 × 10^9^ GC/eye of each vector) were co-injected sub-retinally in 4-week-old C57BL/6 mice with kinase inhibitors at different concentrations (10, 30, and 100 μM). Virus co-injection with PI (30 and 100 μM) served as a positive control. Two months post injection, electroretinographic and optical coherence tomography analyses showed a similar normal retinal function and structure, respectively, independent of treatment (Supplementary Fig. S2). Animals were then sacrificed, and eyecups were harvested, lysed, and analyzed by Western blot with anti-eGFP antibody. Western blot quantification showed that F13 at both 30 and 100 μM resulted in a twofold increment of eGFP expression compared to the DMSO baseline control. On the other hand, PI co-administration resulted in a fourfold increment of eGFP expression compared to DMSO control (data not shown).

**Figure 7.**
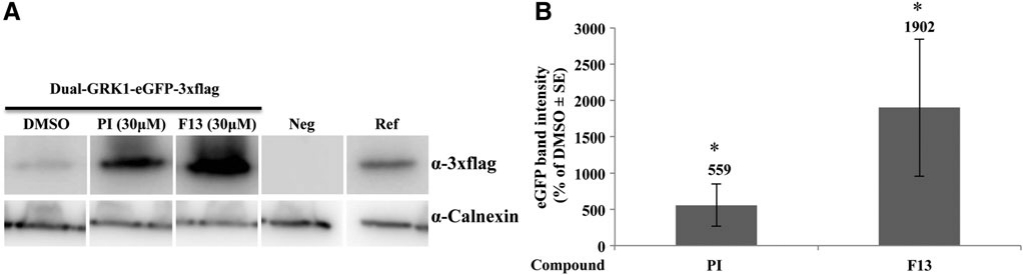
F13 enhances dual AAV8-mediated transduction of the mouse retina. Representative WB image **(A)** and quantification **(B)** of retinal lysates of C57BL/6 eyes co-injected sub-retinally with kinase inhibitors at the indicated concentrations, and dual AAV8 expressing eGFP-3xflag under the control of the PR-specific GRK1 promoter (Dual-GRK1-eGFP-3xflag) or not injected (Neg). For each sample, 65 μg of protein was loaded. Ref, lysate from HEK293 cells transfected with a plasmid expressing eGFP-3xflag used as a reference; α-3xflag, WB with anti-3xflag antibody; α-Calnexin, WB with anti-Calnexin antibody used as loading control; In **(B)**, the values (*n* = 7), normalized to a standard sample present in every WB, are presented as the mean ± SE. *p < 0.05.

Dual AAV8 vector transduction has been reported to be more efficient in the retinal pigmented epithelium than in PR.^26^ To test whether F13 can ameliorate PR transduction, dual AAV8 vectors expressing a 3xflag-tagged eGFP under the control of the PR-specific g-protein couple receptor kinase 1 (GRK1) promoter (dose 5 × 10^9^ GC/eye of each vector) were co-injected sub-retinally in 4-week-old C57BL/6 mice with F13 at a final concentration of 30 μM. Virus co-injection with PI 30 μM served as a positive control. Two months post injection, animals were sacrificed, and retinas were harvested, lysed, and analyzed by Western blot with anti-3xflag antibodies (which are more sensitive than anti-eGFP antibodies; Fig. 7A). Western blot quantification (Fig. 7B) showed that co-administration of F13 resulted in a 19-fold increment of eGFP expression compared to the DMSO control. Importantly, the enhancement obtained with F13 was more than threefold that obtained with PI.

## Discussion

AAV vectors have become increasingly popular for gene therapy of IRDs.^49^ However, their application to diseases affecting the retina and requiring transfer of genes >5 kb (referred to as large genes) is inhibited by the limited cargo capacity of AAV. Several groups, including the authors', have recently reported efficient delivery of large genes to both mouse and pig retina using dual AAV vectors.^22–26^ However, the drawback of this system is that it displays a lower transduction efficiency compared to a single AAV vector.^26^ This could be overcome by increasing the vector dose. However, the sub-retinal space can only accept a limited volume of vector suspension. Alternatively, the transduction efficiency could be increased by positively modulating AAV transduction similarly to what PIs do.^29^

Based on the knowledge that AAV capsid phosphorylation targets AAV particles to degradation^34^ and thus inhibits AAV transduction, HTS was performed of a library of kinase inhibitors, and initially 12 were identified that increase dual AAV2 transduction *in vitro*. However, after validation, five compounds were excluded because they enhanced dual AAV transduction less than the positive control (PI), and four additional compounds were discarded, since they similarly enhanced transgene expression from AAV infection and DNA plasmid transfection, suggesting that the effect of these kinase inhibitors is not specific for AAV transduction. Of the remaining three compounds (F13, K17, and H17) that showed a specific positive effect on AAV2 transduction, K17 had a preferential effect on dual AAV2 vector transduction.

Importantly, the enhancement of dual AAV transduction does not appear to be transgene specific, as a significant increment was observed in three different transgene protein products: eGFP, ABCA4, and MYO7A. While the enhancement in transduction is similar between eGFP and MYO7A, the magnitude of enhancement for ABCA4 was 2.5- to 5-fold lower (Figs. 3 and 4B vs. Fig. 4A). As the drug-induced increase in RNA levels for the three transgenes were similar (Fig. 4A and B), it is possible that the different levels of proteins observed are due to their different stability.

Also, F13, K17, and H17 increase the transduction levels of various AAV serotypes, including AAV1, 2, 8, 9, and rh10 (Figs. 3 and 4C), suggesting that the increase is not serotype dependent. As AAV1, 2, 8, and 9 are known to enter the cells through different receptors,^50^ this suggests that the compounds do not act at this entry step.

It is noteworthy that the enhancement in eGFP transduction was confirmed after knockdown of *AURKA*, *AURKB*, *PLK1*, and *PTK2* (Fig. 5), which are target kinases of F13, K17, and H17, with PTK2 being inhibited by both F13 and H17, which points to the reliability of the screening method.

To shed light on the mechanism involved in kinase inhibitor-mediated enhancement of AAV transduction, early, intermediate, and late steps of AAV entry and transduction were analyzed. In HEK293 cells, α5β1 integrin is an important co-receptor for AAV infection.^46^ The results confirm a direct association between AAV2 VP and β1 integrin. F13, a PTK2 inhibitor, enhances such an association, suggesting that PTK2 phosphorylation negatively affects the kinetic of VP/β1 integrin complex assembly (Fig. 6A). Following entry, AAV capsid protein phosphorylation has been described to target AAV particles to proteasome degradation.^34^ Thus, the study tested if treatment with F13, K17, or H17 decreases AAV capsid phosphorylation. Indeed, a reduction in AAV capsid tyrosine and serine phosphorylation was observed following administration of either K17 or H17 (Fig. 6A). Since both are inhibitors of serine/threonine but not tyrosine kinases, this suggests that their effect on capsid phosphorylation is partly indirect.

Finally, following release of AAV DNA in the nucleus of target cells, the activation of the DNA damage repair mediated by ATM favors the circularization of self-complementary AAV genomes both in *vitro* and *in vivo*.^47^ The study tested whether F13, K17, or H17 induces ATM activation, especially since PLK1, a target of K17, has been reported to inhibit proteins involved in the ATM DNA damage repair pathways such as Mre11^51^ and 53BP1.^52^ ATM activation was observes by H17 but not by K17, suggesting that pathways downstream of AURKA or AURKB may activate ATM (Fig. 6B).

The kinases inhibited by F13, H17, and K17 are not among those that Mano *et al.* found to enhance AAV transduction following siRNA knockdown.^53^ This can be explained by the different cell type used (Hela by Mano *et al.* and HEK293 by the authors). Indeed, dual AAV transduction enhancement was not observed upon infection of Hela cells and addition of the kinase inhibitors selected in the present study (data not shown).

The current *in vivo* experiments in the mouse retina show that neither K17 nor H17 improves dual AAV8 transduction. This might be explained by the low to absent ocular expression of AURKA, AURKB, and PLK1, the main targets of these compounds (www.ebi.ac.uk). However, it is possible that K17 and H17 enhance dual AAV transduction in other tissues where AURKA, AURKB, and PLK1 are expressed at higher levels than the retina. Along a similar line, it was observed that F13 + K17 and H17 + K17 have a synergistic effect *in vitro* (Supplementary Fig. S1), suggesting that these compounds can be used in tissues that co-express their target kinases.

Importantly, while the co-administration of F13 with dual AAV8 vectors with the ubiquitous CMV promoter led to a mild enhancement in efficiency of transduction, the same compound induced a strong enhancement when the transgene was under the control of the PR-specific GRK1 promoter. Noteworthy, the enhancement observed in PR with F13 is 19-fold higher than the one observed in the DMSO baseline control and threefold higher than in the positive control PI (Fig. 7). This is particularly important, as several IRDs, including STGD1 and USH1B, are caused by mutations in genes expressed in PR. The mechanism leading to the observed increase in transduction could be the same as discussed previously. PTK2, the main target of F13, is indeed expressed in the retina, as reported at www.ebi.ac.uk. Interestingly, PTK2 is a target of H17 as well. The lack of effect of this last compound might be explained by the different half maximal inhibitory concentration (IC50) of F13 and H17 for the PTK2 kinase (IC50 F13 = 1.5 nM; IC50 H17 = 20 nM; www.selleckchem.com). Based on this evidence, in the eye, co-administration of F13 with therapeutic single or dual AAV vectors could improve transduction. Alternatively, this may help to reduce the AAV vector dose.

This study only considered kinase inhibitors that enhance AAV transduction, but at the same time, compounds resulting in a decrease of transduction might be equally interesting. Indeed, it could be hypothesized that target kinases of this group of compounds are involved in key steps required for AAV transduction.

In conclusion, taking advantage of state-of-the-art HTS, a robust assay was set up to screen and validate compounds that enhance dual AAV transduction efficiency both *in vitro* and in the retina. This work resulted in the identification of three compounds that significantly and specifically enhance dual AAV2 vector transduction *in vitro*. Moreover, one of these compounds was effective if co-administered with dual AAV8 in the mouse retina. This would favor possible applications of dual AAV for the treatment of IRDs that require the transfer of CDS >5 kb. Finally, this approach is well suited to screen larger libraries of biological modifiers, including FDA-approved drugs, but also siRNA collections, which could lead to further improvement in AAV-based therapy, identification of new target genes to increase AAV transduction, and a better understanding of AAV biology.

## Supplementary Material

Supplemental data

Supplemental data

Supplemental data

Supplemental data

## References

[1] Sohocki MM, Daiger SP, Bowne SJ, et al. Prevalence of mutations causing retinitis pigmentosa and other inherited retinopathies. Hum Mutat 2001;17:42–51 11139241 10.1002/1098-1004(2001)17:1<42::AID-HUMU5>3.0.CO;2-K PMC2585107

[2] Berger W, Kloeckener-Gruissem B, Neidhardt J The molecular basis of human retinal and vitreoretinal diseases. Prog Retin Eye Res 2010;29:335–375 20362068 10.1016/j.preteyeres.2010.03.004

[3] Bainbridge JW, Smith AJ, Barker SS, et al. Effect of gene therapy on visual function in Leber's congenital amaurosis. New Engl J Med 2008;358:2231–2239 18441371 10.1056/NEJMoa0802268

[4] Cideciyan AV, Hauswirth WW, Aleman TS, et al. Vision 1 year after gene therapy for Leber's congenital amaurosis. New Engl J Med 2009;361:725–727 10.1056/NEJMc0903652 PMC2847775 19675341

[5] Maguire AM, Simonelli F, Pierce EA, et al. Safety and efficacy of gene transfer for Leber's congenital amaurosis. New Engl J Med 2008;358:2240–2248 18441370 10.1056/NEJMoa0802315 PMC2829748

[6] Maguire AM, High KA, Auricchio A, et al. Age-dependent effects of RPE65 gene therapy for Leber's congenital amaurosis: a Phase 1 dose-escalation trial. Lancet 2009;374:1597–1605 19854499 10.1016/S0140-6736(09)61836-5 PMC4492302

[7] Simonelli F, Maguire AM, Testa F, et al. Gene therapy for Leber's congenital amaurosis is safe and effective through 1.5 years after vector administration. Mol Ther 2010;18:643–650 19953081 10.1038/mt.2009.277 PMC2839440

[8] Testa F, Maguire AM, Rossi S, et al. Three-year follow-up after unilateral subretinal delivery of adeno-associated virus in patients with Leber congenital amaurosis type 2. Ophthalmology 2013;120:1283–1291 23474247 10.1016/j.ophtha.2012.11.048 PMC3674112

[9] Allocca M, Mussolino C, Garcia-Hoyos M, et al. Novel adeno-associated virus serotypes efficiently transduce murine photoreceptors. J Virol 2007;81:11372–11380 17699581 10.1128/JVI.01327-07 PMC2045569

[10] Mussolino C, della Corte M, Rossi S, et al. AAV-mediated photoreceptor transduction of the pig cone-enriched retina. Gene Ther 2011;18:637–645 21412286 10.1038/gt.2011.3 PMC3131697

[11] Vandenberghe LH, Bell P, Maguire AM, et al. Dosage thresholds for AAV2 and AAV8 photoreceptor gene therapy in monkey. Sci Transl Med 2011;3:88ra54 10.1126/scitranslmed.3002103 PMC5027886 21697530

[12] Auricchio A Fighting blindness with adeno-associated virus serotype 8. Hum Gene Ther 2011;22:1169–1170 22044092 10.1089/hum.2011.2521

[13] Natkunarajah M, Trittibach P, McIntosh J, et al. Assessment of ocular transduction using single-stranded and self-complementary recombinant adeno-associated virus serotype 2/8. Gene Ther 2008;15:463–467 18004402 10.1038/sj.gt.3303074

[14] Boye SE, Alexander JJ, Boye SL, et al. The human rhodopsin kinase promoter in an AAV5 vector confers rod- and cone-specific expression in the primate retina. Hum Gene Ther 2012;23:1101–1115 22845794 10.1089/hum.2012.125 PMC3472519

[15] Vandenberghe LH, Bell P, Maguire AM, et al. AAV9 targets cone photoreceptors in the nonhuman primate retina. PloS One 2013;8:e53463 23382846 10.1371/journal.pone.0053463 PMC3559681

[16] Lai Y, Yue Y, Duan D Evidence for the failure of adeno-associated virus serotype 5 to package a viral genome > or = 8.2 kb. Mol Ther 2010;18:75–79 19904238 10.1038/mt.2009.256 PMC2839223

[17] Hermonat PL, Quirk JG, Bishop BM, et al. The packaging capacity of adeno-associated virus (AAV) and the potential for wild-type-plus AAV gene therapy vectors. FEBS Lett 1997;407:78–84 9141485 10.1016/s0014-5793(97)00311-6

[18] Dong B, Nakai H, Xiao W Characterization of genome integrity for oversized recombinant AAV vector. Mol Ther 2010;18:87–92 19904236 10.1038/mt.2009.258 PMC2803017

[19] Wu Z, Yang H, Colosi P Effect of genome size on AAV vector packaging. Mol Ther 2010;18:80–86 19904234 10.1038/mt.2009.255 PMC2839202

[20] Allikmets R A photoreceptor cell-specific ATP-binding transporter gene (ABCR) is mutated in recessive Stargardt macular dystrophy. Nat Genet 1997;17:122 10.1038/ng0997-122a 9288113

[21] Millan JM, Aller E, Jaijo T, et al. An update on the genetics of usher syndrome. J Ophthalmol 2011;2011:417217 21234346 10.1155/2011/417217 PMC3017948

[22] Yan Z, Zhang Y, Duan D, et al. Trans-splicing vectors expand the utility of adeno-associated virus for gene therapy. Proc Natl Acad Sci U S A 2000;97:6716–6721 10841568 10.1073/pnas.97.12.6716 PMC18714

[23] Duan D, Yue Y, Engelhardt JF Expanding AAV packaging capacity with trans-splicing or overlapping vectors: a quantitative comparison. Mol Ther 2001;4:383–391 11592843 10.1006/mthe.2001.0456

[24] Ghosh A, Duan D Expanding adeno-associated viral vector capacity: a tale of two vectors. Biotechnol Genet Eng Rev 2007;24:165–177 18059632 10.1080/02648725.2007.10648098

[25] Ghosh A, Yue Y, Lai Y, et al. A hybrid vector system expands adeno-associated viral vector packaging capacity in a transgene-independent manner. Mol Ther 2008;16:124–130 17984978 10.1038/sj.mt.6300322

[26] Trapani I, Colella P, Sommella A, et al. Effective delivery of large genes to the retina by dual AAV vectors. EMBO Mol Med 2014;6:194–211 24150896 10.1002/emmm.201302948 PMC3927955

[27] Reich SJ, Auricchio A, Hildinger M, et al. Efficient trans-splicing in the retina expands the utility of adeno-associated virus as a vector for gene therapy. Hum Gene Ther 2003;14:37–44 12573057 10.1089/10430340360464697

[28] Knipe DM, Howley PM Fields Virology. Philadelphia, PA: Wolters Kluwer/Lippincott Williams & Wilkins Health, 2013

[29] Duan D, Yue Y, Yan Z, et al. Endosomal processing limits gene transfer to polarized airway epithelia by adeno-associated virus. J Clin Invest 2000;105:1573–1587 10841516 10.1172/JCI8317 PMC300848

[30] Jennings K, Miyamae T, Traister R, et al. Proteasome inhibition enhances AAV-mediated transgene expression in human synoviocytes *in vitro* and *in vivo*. Mol Ther 2005;11:600–607 15771962 10.1016/j.ymthe.2004.10.020

[31] Lopes VS, Boye SE, Louie CM, et al. Retinal gene therapy with a large MYO7A cDNA using adeno-associated virus. Gene Ther 2013;20:824–833 23344065 10.1038/gt.2013.3 PMC3640772

[32] Nicolson SC, Li C, Hirsch ML, et al. Identification and validation of small molecules that enhance recombinant adeno-associated virus transduction following high-throughput screens. J Virol 2016;90:7019–7031 27147738 10.1128/JVI.02953-15 PMC4984620

[33] Zhong L, Zhao W, Wu J, et al. A dual role of EGFR protein tyrosine kinase signaling in ubiquitination of AAV2 capsids and viral second-strand DNA synthesis. Mol Ther 2007;15:1323–1330 17440440 10.1038/sj.mt.6300170

[34] Zhong L, Li B, Jayandharan G, et al. Tyrosine-phosphorylation of AAV2 vectors and its consequences on viral intracellular trafficking and transgene expression. Virology 2008;381:194–202 18834608 10.1016/j.virol.2008.08.027 PMC2643069

[35] Petrs-Silva H, Dinculescu A, Li Q, et al. High-efficiency transduction of the mouse retina by tyrosine-mutant AAV serotype vectors. Mol Ther 2009;17:463–471 19066593 10.1038/mt.2008.269 PMC2835095

[36] Aslanidi GV, Rivers AE, Ortiz L, et al. Optimization of the capsid of recombinant adeno-associated virus 2 (AAV2) vectors: the final threshold? PloS One 2013;8:e59142 23527116 10.1371/journal.pone.0059142 PMC3602601

[37] Auricchio A, Hildinger M, O'Connor E, et al. Isolation of highly infectious and pure adeno-associated virus type 2 vectors with a single-step gravity-flow column. Hum Gene Ther 2001;12:71–76 11177544 10.1089/104303401450988

[38] Doria M, Ferrara A, Auricchio A AAV2/8 vectors purified from culture medium with a simple and rapid protocol transduce murine liver, muscle, and retina efficiently. Hum Gene Ther Methods 2013;24:392–398 24116943 10.1089/hgtb.2013.155 PMC3869536

[39] Drittanti L, Rivet C, Manceau P, et al. High throughput production, screening and analysis of adeno-associated viral vectors. Gene Ther 2000;7:924–929 10849551 10.1038/sj.gt.3301191

[40] Mukaka MM Statistics corner: a guide to appropriate use of correlation coefficient in medical research. Malawi Med J 2012;24:69–71 23638278 PMC3576830

[41] Castoria G, Giovannelli P, Lombardi M, et al. Tyrosine phosphorylation of estradiol receptor by Src regulates its hormone-dependent nuclear export and cell cycle progression in breast cancer cells. Oncogene 2012;31:4868–4877 22266855 10.1038/onc.2011.642

[42] Liang FQ, Anand V, Maguire AM, et al. Intraocular delivery of recombinant virus. Methods Mol Med 2001;47:125–139 21394582 10.1385/1-59259-085-3:125

[43] Benjamini Y and Hochberg Y Controlling the false discovery rate: a practical and powerful approach to multiple testing. J R Stat Soc B Methodol 1995;57:289–300

[44] Kalaitzis AA, Lawrence ND A simple approach to ranking differentially expressed gene expression time courses through Gaussian process regression. BMC Bioinformatics 2011;12:180 21599902 10.1186/1471-2105-12-180 PMC3116489

[45] Gao G, Vandenberghe LH, Alvira MR, et al. Clades of adeno-associated viruses are widely disseminated in human tissues. J Virol 2004;78:6381–6388 15163731 10.1128/JVI.78.12.6381-6388.2004 PMC416542

[46] Asokan A, Hamra JB, Govindasamy L, et al. Adeno-associated virus type 2 contains an integrin alpha5beta1 binding domain essential for viral cell entry. J Virol 2006;80:8961–8969 16940508 10.1128/JVI.00843-06 PMC1563945

[47] Choi VW, McCarty DM, Samulski RJ Host cell DNA repair pathways in adeno-associated viral genome processing. J Virol 2006;80:10346–10356 17041215 10.1128/JVI.00841-06 PMC1641795

[48] Rose JA, Maizel JV Jr, Inman JK, et al. Structural proteins of adenovirus-associated viruses. J Virol 1971;8:766–770 5132697 10.1128/jvi.8.5.766-770.1971 PMC376258

[49] Colella P, Auricchio A Gene therapy of inherited retinopathies: a long and successful road from viral vectors to patients. Hum Gene Ther 2012;23:796–807 22734691 10.1089/hum.2012.123 PMC3413895

[50] Nonnenmacher M, Weber T Intracellular transport of recombinant adeno-associated virus vectors. Gene Ther 2012;19:649–658 22357511 10.1038/gt.2012.6 PMC4465241

[51] Li Z, Li J, Kong Y, et al. Plk1 Phosphorylation of Mre11 antagonizes the DNA damage response. Cancer Res 2017;77:3169–3180 28512243 10.1158/0008-5472.CAN-16-2787 PMC5504882

[52] Benada J, Burdova K, Lidak T, et al. Polo-like kinase 1 inhibits DNA damage response during mitosis. Cell Cycle 2015;14:219–231 25607646 10.4161/15384101.2014.977067 PMC4613155

[53] Mano M, Ippodrino R, Zentilin L, et al. Genome-wide RNAi screening identifies host restriction factors critical for *in vivo* AAV transduction. Proc Natl Acad Sci U S A 2015;112:11276–11281 26305933 10.1073/pnas.1503607112 PMC4568673

[54] Roberts WG, Ung E, Whalen P, et al. Antitumor activity and pharmacology of a selective focal adhesion kinase inhibitor, PF-562,271. Cancer Res 2008;68:1935–1944 18339875 10.1158/0008-5472.CAN-07-5155

[55] Jani JP, Arcari J, Bernardo V, et al. PF-03814735, an orally bioavailable small molecule aurora kinase inhibitor for cancer therapy. Mol Cancer Ther 2010;9:883–894 20354118 10.1158/1535-7163.MCT-09-0915

[56] Steegmaier M, Hoffmann M, Baum A, et al. BI 2536, a potent and selective inhibitor of polo-like kinase 1, inhibits tumor growth *in vivo*. Curr Biol 2007;17:316–322 17291758 10.1016/j.cub.2006.12.037

[57] Heinrich MC, Griffith D, McKinley A, et al. Crenolanib inhibits the drug-resistant PDGFRA D842V mutation associated with imatinib-resistant gastrointestinal stromal tumors. Clin Cancer Res 2012;18:4375–4384 22745105 10.1158/1078-0432.CCR-12-0625

[58] Wernig G, Kharas MG, Okabe R, et al. Efficacy of TG101348, a selective JAK2 inhibitor, in treatment of a murine model of JAK2V617F-induced polycythemia vera. Cancer Cell 2008;13:311–320 18394554 10.1016/j.ccr.2008.02.009

[59] Anderson K, Lai Z, McDonald OB, et al. Biochemical characterization of GSK1070916, a potent and selective inhibitor of aurora B and aurora C kinases with an extremely long residence time. Biochem J 2009;420:259–265 19284385 10.1042/BJ20090121

[60] Braselmann S, Taylor V, Zhao H, et al. R406, an orally available spleen tyrosine kinase inhibitor blocks fc receptor signaling and reduces immune complex-mediated inflammation. J Pharmacol Exp Ther 2006;319:998–1008 16946104 10.1124/jpet.106.109058

