# Supplementary material for: High-Throughput Screening Identifies Kinase Inhibitors That Increase Dual Adeno-Associated Viral Vector Transduction In Vitro and in Mouse Retina
Source: Hum Gene Ther. 2018 Aug 1;29(8):886–901. doi: 10.1089/hum.2017.220 (PMC6098407; doi:10.1089/hum.2017.220)
Supplement: Supplemental data [file Supp_Fig1.pdf]

Supplementary Data

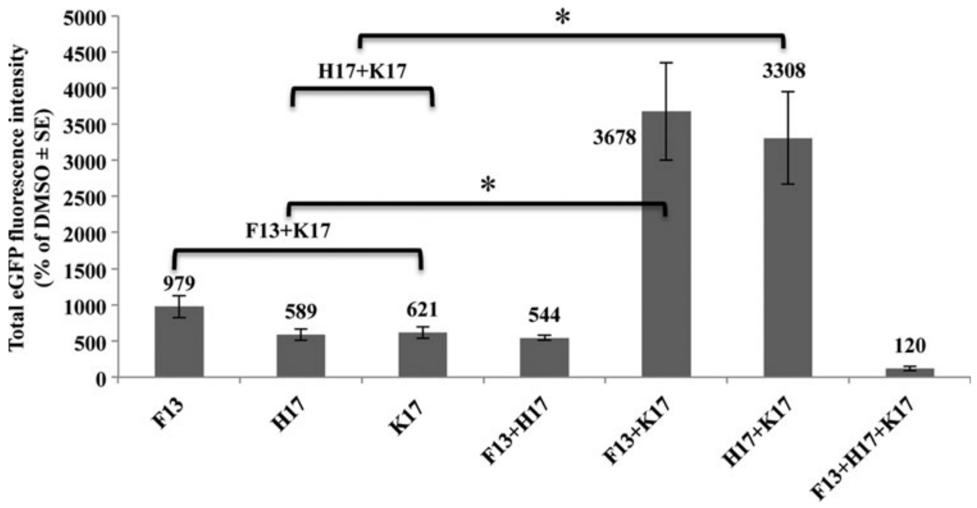

Supplementary Figure S1. Synergistic effect of F13 + K17 and H17 + K17. Cytofluorimetric analysis of eGFP expression in HEK293 cells infected with dual AAV2 expressing eGFP and incubated with the various drugs either alone or in combination. Values ($n=3$), normalized to the corresponding DMSO sample, are presented as the mean $\pm$ SE. Drug concentrations: F13 5 μM, H17 3 μM, and K17 0.3 μM. *$p \leq 0.05$. AAV2, dual adeno-associated viral serotype 2; DMSO, dimethyl sulfoxide; eGFP, enhanced green fluorescent protein.
