# Supplementary material for: High-Throughput Screening Identifies Kinase Inhibitors That Increase Dual Adeno-Associated Viral Vector Transduction In Vitro and in Mouse Retina
Source: Hum Gene Ther. 2018 Aug 1;29(8):886–901. doi: 10.1089/hum.2017.220 (PMC6098407; doi:10.1089/hum.2017.220)
Supplement: Supplemental data [file Supp_Fig2.pdf]

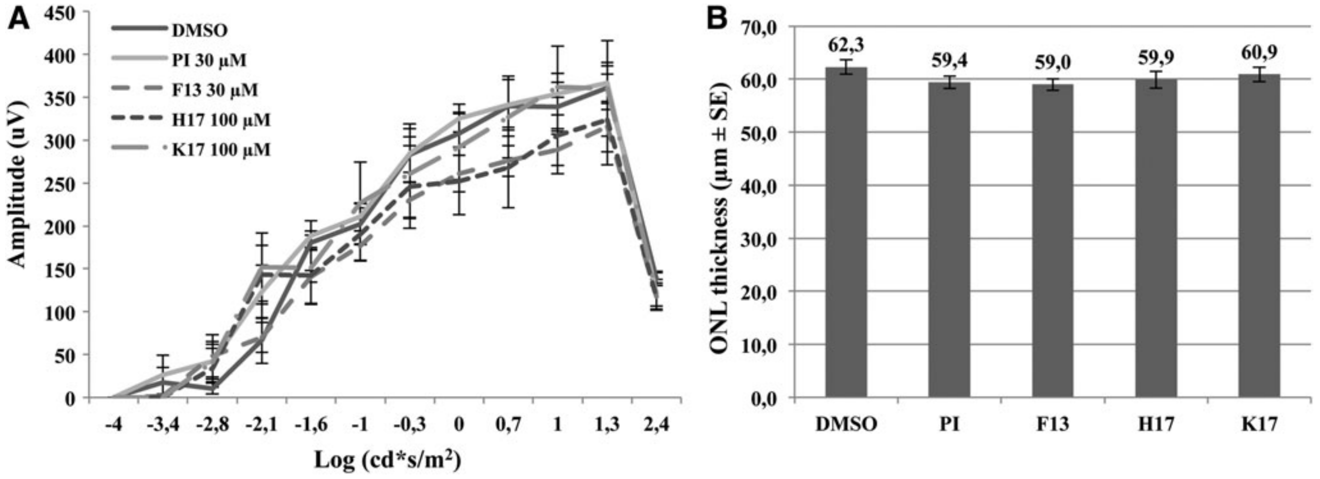

Supplementary Figure S2. Absence of detectable toxicity upon sub-retinal administration of dual AAV8 and the kinase inhibitors in mice. **(A** and **B)** Electroretinographic analysis of a-wave light responses **(A)** and Spectral domain optical coherence tomogram analysis **(B)** of C57BL/6 mice co-injected sub-retinally with dual AAV8 vectors and the indicated drugs. Results ($n = 6$) are reported as the mean ± SE. Light intensity of 20 cd s/m^2 (a-wave), background white light of 50 cd s/m^2, and light intensity of 20 cd s/m^2 (b-wave). Drug concentrations: PI 30 μM, F13 30 μM, H17 100 μM, and K17 100 μM.
