# Supplementary material for: High-Throughput Screening Identifies Kinase Inhibitors That Increase Dual Adeno-Associated Viral Vector Transduction In Vitro and in Mouse Retina
Source: Hum Gene Ther. 2018 Aug 1;29(8):886–901. doi: 10.1089/hum.2017.220 (PMC6098407; doi:10.1089/hum.2017.220)
Supplement: Supplemental data [file Supp_Fig3.pdf]

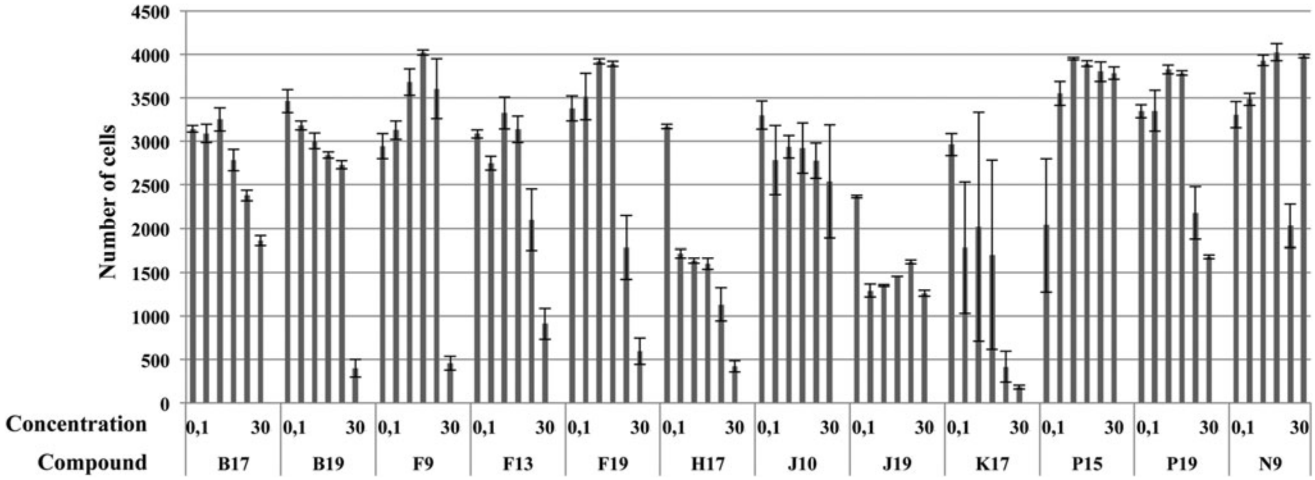

Supplementary Figure S3. Cell viability upon exposure to various doses of the kinase inhibitors. HEK293 cells, infected at $t=24$ h with dual AAV2-eGFP vectors (MOI $=5\times10^4$ each vector), were incubated with the indicated compounds at doses ranging from 0.1 to 30 μM (0.1, 0.3, 1, 3, 10, and 30 μM). Surviving cells were counted with the OPERA system 72 h after the treatment. Values ($n=2$) are presented as the mean $\pm$ SE. MOI, multiplicity of infection.
