# Supplementary material for: High-Throughput Screening Identifies Kinase Inhibitors That Increase Dual Adeno-Associated Viral Vector Transduction In Vitro and in Mouse Retina
Source: Hum Gene Ther. 2018 Aug 1;29(8):886–901. doi: 10.1089/hum.2017.220 (PMC6098407; doi:10.1089/hum.2017.220)
Supplement: Supplemental data [file Supp_Fig4.pdf]

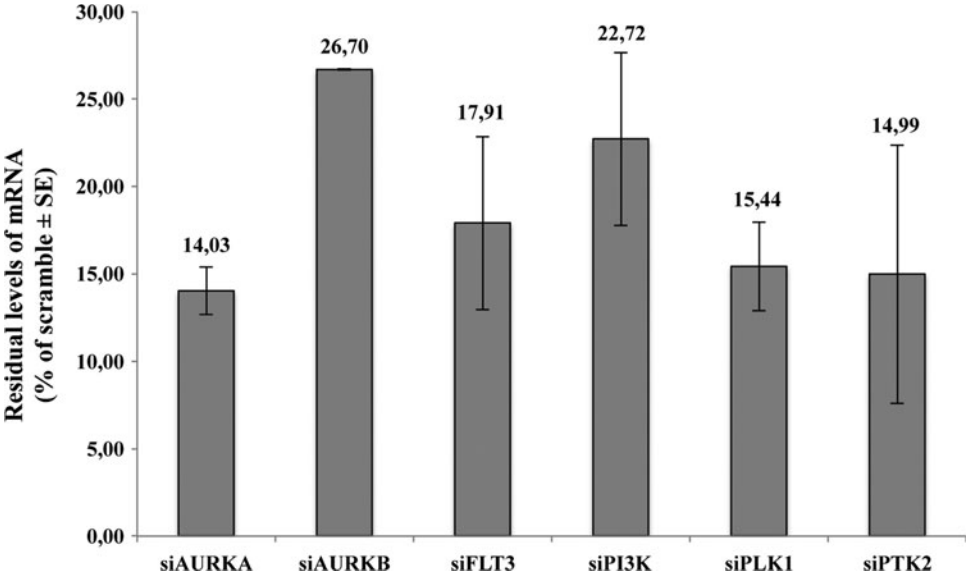

Supplementary Figure S4. SiRNA mediated knockdown of the selected kinases. Residual levels of kinase mRNA ($n=2$) were calculated using the $\Delta\Delta Cq$ formula. Results are expressed as the mean ± SE.
